# Single injection of lidocaine to reduce tourniquet hypertension in ambulatory arthroscopic patients under general anaesthesia: randomized, double-blind, placebo-controlled clinical trial

**DOI:** 10.1093/bjsopen/zrad014

**Published:** 2023-04-28

**Authors:** Yue Sun, Jianhui Zhao, Wenli Guo, Hailing Mu, Ying Zhao, Yaping Xie, Chen Yang, Yao Lu

**Affiliations:** Department of Anesthesiology, The First Affiliated Hospital of Anhui Medical University, Hefei, China; Department of Anesthesiology, The First Affiliated Hospital of Anhui Medical University, Hefei, China; Department of Anesthesiology, The First Affiliated Hospital of Anhui Medical University, Hefei, China; Department of Anesthesiology, The First Affiliated Hospital of Anhui Medical University, Hefei, China; Department of Anesthesiology, The First Affiliated Hospital of Anhui Medical University, Hefei, China; Department of Anesthesiology, The First Affiliated Hospital of Anhui Medical University, Hefei, China; Department of Anesthesiology, The First Affiliated Hospital of Anhui Medical University, Hefei, China; Department of Anesthesiology, The First Affiliated Hospital of Anhui Medical University, Hefei, China; Ambulatory Surgery Center, The First Affiliated Hospital of Anhui Medical University, Hefei, China

## Abstract

**Background:**

This prospective study investigated whether a single dose of intravenous lidocaine could alleviate tourniquet hypertension in patients undergoing ambulatory arthroscopy under general anaesthesia.

**Methods:**

Patients aged 18–65 years undergoing knee arthroscopy under general anaesthesia were randomly divided into the lidocaine group (L group) and the normal saline group (N group). Patients received an intravenous injection 10 min before tourniquet inflation of either 1.5mg/kg lignocaine made up to 10ml with 0.9 per cent normal saline, or 10ml of 0.9 per cent normal saline. The primary outcome was the incidence of tourniquet hypertension. Secondary outcomes included haemodynamic changes, degree of elevation of blood pressure, changes in serum inflammatory indicators including interleukin 6 and tumour necrosis factor-α, the numerical rating scale, 15-item quality of recovery after surgery, the incidence of adverse events and the duration of hospital stay. Randomization was computer-generated with allocation concealment by sealed envelopes. Patients, caregivers and researchers were all blind to the allocation group throughout the study.

**Results:**

Ninety-six patients were included in the study; 48 in each group. Compared with the N group, the incidence of tourniquet hypertension in the L group was significantly lower (37.5 per cent *versus* 68.8 per cent; *P* < 0.002). The degree of elevation of systolic blood pressure from baseline to the end of surgery in the L group was significantly lower than the N group (17.1 per cent *versus* 23.6 per cent; *P* = 0.020). The concentration of tumour necrosis factor-α in the L group 5 min after tourniquet deflation was lower than in the N group (32.12 pg/ml *versus* 39.89 pg/ml; *P* = 0.029). The median numerical rating scale of the L group was significantly lower at 6 h (0 *versus* 3.0; *P* = 0.003) and 24 h (0 *versus* 2.0; *P* < 0.001) after surgery. In the L group, the total 15-item quality of recovery was significantly increased (131 *versus* 128; *P* = 0.017).

**Conclusion:**

Single injection of intravenous lidocaine alleviated tourniquet hypertension in ambulatory arthroscopic patients under general anaesthesia. Intravenous lidocaine can inhibit tourniquet hypertension formation by reducing tumour necrosis factor-α release, and has beneficial effects on postoperative pain and recovery.

**Registration number:**

ChiCTR2200055551 (http://www.chictr.org.cn/edit.aspx? pid=148235&htm=4).

## Teaser

Single injection of lidocaine alleviates tourniquet hypertension in daytime arthroscopic patients under general anaesthesia. Intravenous lidocaine can inhibit tourniquet hypertension formation by reducing tumour necrosis factor-α release, and has beneficial effects on postoperative pain and recovery quality.

## Introduction

Knee pain is very common and ambulatory knee arthroscopic surgery has increased significantly^1^. Ambulatory surgery is performed in a planned, non-hospitalization situation with admission, surgery and departure completed within 24 h. Ambulatory knee arthroscopic surgery is mainly aimed at meniscus injury, ligament injury and synovitis^2^. Patients suffering from arthropathy not only have less surgical trauma and shorter duration of hospital stay, but also recover safely and quickly which reduces the hospitalization cost^3^.

Using a tourniquet for the affected limb is an important step in the preoperative preparation for knee arthroscopic surgery under general anaesthesia. Surgeons place the tourniquet cuff at the root of the thigh, raise the affected limb, block the blood flow with an elastic bandage, then inflate the tourniquet^4^. The tourniquet can improve surgical vision, reduce blood loss, shorten the surgery time and improve antibiotic infusion^5^. Despite these advantages, intraoperative tourniquet hypertension (TH) is an obvious disadvantage^6^. A retrospective study reported that 67 per cent of patients undergoing orthopaedic surgery under general anaesthesia using a tourniquet developed TH^7^.

TH, also called ‘tourniquet pain’, occurs when 30–60 min after the tourniquet is inflated under general anaesthesia, the patient may have increased systolic or diastolic blood pressure (SBP or DBP), accompanied by an increase in heart rate (HR), which will continue until the tourniquet deflates. TH is considered to have occurred when the SBP or DBP is ≥ 30 per cent higher than the baseline (1–2 min after tourniquet inflation or skin incision)^7,8^. TH leads to postoperative pain, increases the risk of nerve, muscle and vascular injury, and affects the postoperative rehabilitation of patients^9^. Although many studies have investigated this problem, most therapeutic effects are not satisfactory^10–13^. The pathogenesis of TH may be that local compression causes chronic pain mediated by unmyelinated and slow conducting C fibres^7,14^. Neuroinflammation may be the key pathological process of TH. A tourniquet induces limb ischaemia, which can promote a proinflammatory state, activate the production of inflammatory mediators, drive the inflammatory signal cascade, lead to the activation of nociceptors, resulting in chronic pain^15,16^.

Lidocaine is an amino amide-type local anaesthetic. In recent years, randomized reports have shown that perioperative intravenous lidocaine (IVL), as part of multimodal pain treatment, has antiarrhythmia effects, significantly reduces spontaneous pain, abnormal pain or hyperalgesia, reduces pain and opioid demand within 24 h after surgery, improves rehabilitation and shortens the duration of hospital stay^17–19^. Lidocaine also reduces the release of inflammatory mediators such as interleukin 1 and 6 (IL-1, IL-6), and tumour necrosis factor-α (TNF-α)^17^. Based on prior studies, this trial aimed to explore whether a single dose of IVL could reduce the incidence of TH in patients undergoing ambulatory knee arthroscopy under general anaesthesia, and whether IVL could reduce the release of inflammatory factors and reduce hyperalgesia.

## Methods

### Study design and participants

A randomized, double-blind, placebo-controlled clinical trial was conducted in the ambulatory surgery centre of the First Affiliated Hospital of Anhui Medical University. The trial plan was approved by the ethics committee of the First Affiliated Hospital of Anhui Medical University (PJ2022-02-34). The study was registered in the Chinese Clinical Trial Registry (ChiCTR220055551, Principal Investigator: Y.L.) on 12 January 2022. Written informed consent was obtained from each patient and the study took place between 11 February 2022 and 20 June 2022.

Inclusion criteria included: age 18–65 years, ASA classification I–II, BMI of 18–30 kg/m^2^, undergoing ambulatory knee arthroscopy under general anaesthesia using tourniquet. Exclusion criteria included: history of hypertension, peripheral vascular disease, diabetes; history of deep vein thrombosis; history of heart, liver or kidney disease; history of allergic reaction to amide local anaesthetics; history of cerebral infarction or seizures; history of dependence on analgesic drugs (opioid dependence or non-steroidal anti-inflammatory drugs); history of psychiatric disorders such as schizophrenia, depression or dementia; unable to understand the 15-item quality of recovery (QoR-15) or pain numerical rating scales (NRS). Patients with surgery time of less than 30 min or more than 2 h, patients who had serious adverse events undergoing surgery or were transferred to ICU, and those with incomplete follow-up data were also excluded from the trial.

### Randomization and blinding

A research statistician used a computer to generate random numbers. The randomization code was contained in a sealed envelope. It was sent to the researcher by the research nurse the day before the surgery. An anaesthetist evaluated the suitability for inclusion of patients before surgery, recruited patients, received random codes from the researcher, then assigned patients to the two groups. The study drugs included a single dose of IVL with a concentration of 1.6 per cent (the dosage was 1.5 mg/kg, diluted to 10 ml with 0.9 per cent normal saline) and 0.9 per cent normal saline (10 ml), which were packed in containers with the same colour and packaging. Patients were randomly assigned to the lidocaine group (L group) or the normal saline group (N group) in a ratio of 1 : 1. Throughout the study, patients, researchers, anaesthetists, surgeon, nurses in the postanaesthesia nursing unit (PACU) and wards, and perioperative observation index recorders were all blind to the allocation of the study group.

### Study treatments

All patients in this study were admitted on the day of surgery and discharged on the first day after surgery, during which one overnight stay was required. The patients received routine preoperative preparation and no premedication was administered. Standardized vital signs monitoring, including noninvasive BP monitoring, ECG, pulse oxygen saturation (SpO_2_), bispectral index (BIS) monitoring were implemented. The invasive BP and blood gas analysis was monitored when necessary. To facilitate laryngeal mask intubation, sufentanil (0.4 μg/kg), propofol (2.0 mg/kg), *cis*-atracurium (0.2–0.4 mg/kg) were administered intravenously. Propofol (4–6 mg per kg per h) and remifentanil (0.2–0.5 μg per kg per min) were used for anaesthesia maintenance. To maintain adequate muscle relaxation, *cis*-atracurium (0.2 mg/kg) was given intermittently. The concentration of propofol was adjusted to maintain the BIS value in the range of 40–60.

The non-invasive BP monitoring instrument regularly measured the patient's BP every 5 min; ECG continuously monitored the patient's HR. When the measured BP fluctuated more than 20 per cent of the baseline level, the BP was measured again immediately. If it still fluctuated more than 20 per cent of the baseline level, sufentanil (5–10 μg) was given. The BP was measured again 5 min after sufentanil was given. If the BP fluctuation was lower than 20 per cent of the baseline level, the haemodynamic changes were continuously monitored and observed. No additional sufentanil was used during the surgery. Hypotension (the BP was decreased by more than 20 per cent of baseline) was treated with ephedrine (0.6 mg) and bradycardia (HR of < 45 beats/min) with atropine (0.5 mg). Hypertension (SBP > 150 mmHg) was treated with nicardipine (5 μg/kg) and tachycardia (HR > 90 beats/min) with esmolol (0.3 mg/kg).

Ten minutes before tourniquet inflation, the L group was administered a single-dose IVL with a concentration of 1.6 per cent lidocaine (1.5 mg/kg), diluted to 10 ml with 0.9 per cent normal saline, with an administration rate of 1 ml/s. The N group was injected with 10 ml 0.9 per cent normal saline intravenously at the same time.

Afterwards the surgeon placed the tourniquet on the upper thigh of the operative side, raised the limb to 45°, then used an elastic bandage to help reduce the amount of blood accumulated in the leg. The tourniquet cuff was then inflated by the surgeon to 250 mmHg. After the tourniquet was inflated, the surgeons immediately performed a skin cutting surgery (the surgery began); the tourniquet was deflated immediately after the skin suture (the surgery ended). Therefore, the duration of tourniquet inflation was equal to the duration of surgery. Remifentanil infusion was stopped at the end of the skin suture and the propofol infusion was stopped after the tourniquet was deflated.

After the surgery, each patient was administered 50 mg flurbiprofen axetil for analgesia, then sent to PACU. When autonomous breathing was regular, tidal volume was > 6 ml/kg, SpO_2_ could be maintained at more than 95 per cent during air intake, the laryngeal mask was withdrawn and a Ramsay sedation score (RSS) was performed. Postoperative analgesia pumps were not used. If patients complained that analgesics or antiemetics were needed within 24 h after surgery or the patient vomited more than twice, flurbiprofen axetil (50 mg) or metoclopramide hydrochloride (10–20 mg) were used respectively.

### Outcomes

The primary outcome was the incidence of TH. Secondary outcomes included haemodynamic changes at each time point, degree of elevations of BP, IL-6 and TNF-α concentration change in venous blood at each time point, NRS at each time point after surgery, QoR-15 score at 24 h after surgery, the incidence of adverse events and the duration of hospital stay.

TH was defined as a 30 per cent increase in SBP or DBP from baseline undergoing tourniquet use^7^. As BP may change before surgery (because tension leads to increased BP) and may decrease after anaesthesia induction (drug action leads to decreased BP), this study chose the first measurement reading 2 min after tourniquet inflation as the baseline value (because skin cutting was usually carried out immediately after tourniquet inflation, it was also 2 min after skin cutting)^8^. At the same time, SBP, DBP, mean arterial pressure (MAP) and HR were recorded separately before entering the operating room (T_0_), 2 min after the tourniquet was inflated (baseline, T_1_), 30 min after the tourniquet was inflated (T_2_), the surgery ended or the tourniquet was deflated (T_3_), and 5 min after the tourniquet was deflated (T_4_).

Moreover, NRS was used to assess the pain of patients before surgery and 1 h, 6 h, 24 h after surgery (0, no pain; 10, the most severe pain)^20^. QoR-15 was used to assess the quality of recovery before and 24 h after surgery (five dimensions: emotional state (four items), physical comfort (five items), psychological support (two items), physical independence (two items) and pain (two items))^21^. The total score of QoR-15 ranges from 0 (the worst recovery quality) to 150 (the best recovery quality). In the PACU, RSS was used to assess sedation levels (1, agitated; 2, cooperative and oriented; 3, can respond to simple questions; 4, asleep, but with a quick reaction to stimulus; 5, asleep, arousable; 6, asleep, unarousable)^22^. The patients were asked whether there were adverse reactions such as postoperative nausea and vomiting (PONV), dizziness, hypotension, hypertension, urination pain at 1, 6 and 24 h after surgery. Only the answer of ‘Yes’ or ‘No’ could be accepted.

Patients’ venous blood (5 ml) was drawn before anaesthesia induction (T_5_), 30 min after tourniquet inflation (T_2_) and 5 min after tourniquet deflation (T_4_)^16,23^. Biomarkers that may be related to inflammatory reaction were measured by enzyme-linked immunosorbent assay. The blood was maintained at room temperature for 30 min. Serum was obtained by centrifuging blood at 4000*g* for 20 min at 4°C and storing at −80°C. IL-6 and TNF-α were determined by enzyme-linked immunosorbent assay kit-α concentration^24^.

### Statistical analysis

Based on the preliminary trial, it was observed that approximately 70 per cent of patients developed TH during knee arthroscopy after 10 ml of 0.9 per cent normal saline was given. A 30 per cent decline in the occurrence of TH was considered clinically significant; hence, 43 patients were required per group with an alpha level of 0.05 and power of 90 per cent. Considering a 20 per cent dropout, the aim was to enrol 108 patients.

Categorical data are presented as number (percentage) and were analysed by the chi-square test or Fisher’s exact test. The independent *t* test or Mann–Whitney *U* test was applied for analysing continuous variables, which are presented as mean ± standard deviation or medians (interquartile range). The χ^2^ test or Fisher’s exact test was used to analyse the incidences of TH and adverse events presented as number (percentage). The haemodynamic data and postoperative NRS score were measured repeatedly by ANOVA. For repeated outcome measurements, the *P* values were corrected using the Bonferroni–Holm adjustment. The degree of elevations of SBP and DBP at the end of surgery (percentage of baseline value) was calculated and analysed as the continuous variable. Two-tailed *P* < 0.05 was considered statistically significant. Statistical analyses were performed with SPSS version 25 and GraphPad Prism version 6.

## Results

### Patient characteristics

**Fig. 1:**
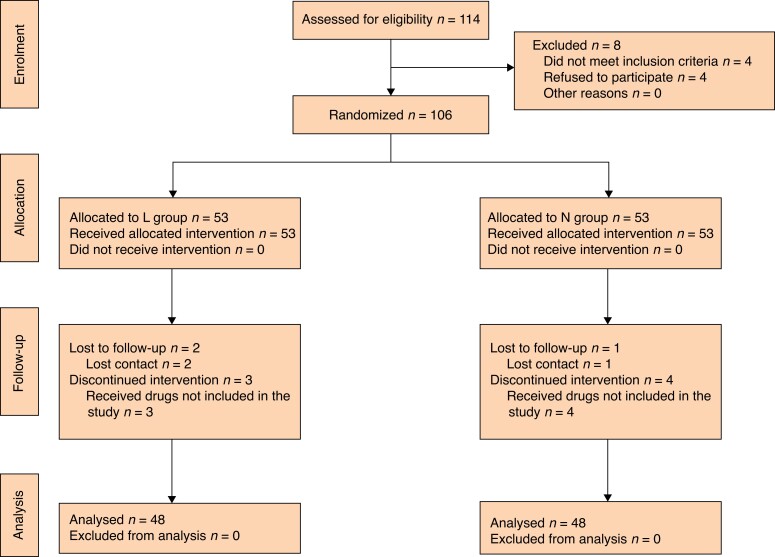
CONSORT flow diagram L group, lidocaine group; N group, normal saline group.

From 11 February 2022 to 20 June 2022, a total of 114 patients were screened for this study. Among them, four patients didn't meet the inclusion criteria and four patients refused to participate in the trial. Therefore, 106 subjects were enrolled. Among the enrolled patients, seven patients were excluded because of incompletion of the intervention (receiving drugs outside the protocol of the study) and three patients were lost to follow-up (lost contact). In total, 96 patients finished the study: 48 in the L group and 48 in the N group (*Fig. 1*).

*Table 1* shows the baseline characteristics of patients and a good balance was maintained between the two groups. No significant differences were found in baseline characteristics, smoking status, history of motion sickness or preoperative nausea and vomiting between the two groups. In this study, the most frequent type of surgery was meningoplasty (59.4 per cent), followed by meningoplasty combined with meniscus suture (35.4 per cent), anterior cruciate ligament reconstruction (ACLR) (4.2 per cent) and one patient received fixation of a tibial intercondylar spinal fracture (1.0 per cent). No significant differences were found in intraoperative variables including anaesthesia time, surgery/tourniquet inflation time, time from anaesthesia induction to IVL administration, time from T_2_ to T_3_ and consumption of anaesthetic drugs between groups. However, in the N group, two patients were given nicardipine because of SBP > 150 mmHg (*P* = 0.241) and three patients were given esmolol because of HR > 90 beats/min (*P* = 0.475) (*Table 1*).

**Table 1:** Patient baseline characteristics and intraoperative characteristics

Baseline characteristics/preoperative score	L group (*n* = 48)	N group (*n* = 48)	*P*
**Median age (i.q.r.), years**	47 (31-52)	44 (32-51)	0.500*
**Mean (s.d.) BMI, kg/m^2^**	23.90 (2.87)	24.07 (2.23)	0.753†
**Sex**			0.221†
Male	27 (56.2)	21 (43.8)	
Female	21 (43.8)	27 (56.2)	
**ASA classification**			
I	14 (29.2)	17 (35.4)	0.513†
II	34 (70.8)	31 (64.6)	
**Baseline characteristics**			
Current smoker	14 (29.2)	12 (25.0)	0.646†
History of motion sickness	10 (20.8)	16 (33.3)	0.168†
Preoperative nausea and vomiting.	0 (0)	2 (4.2)	0.475‡
**Surgery**			
Meningoplasty	27 (56.2)	30 (62.5)	0.533†
Meningoplasty and suture	18 (37.5)	16 (33.3)	0.670†
ACLR	3 (6.3)	1 (2.1)	0.610‡
Fixation of tibial intercondylar spine fracture	0 (0)	1 (2.1)	1.000‡
**Median duration (i.q.r.) (min)**			
Anaesthesia	83 (74-93)	87 (75-96)	0.179*
Surgery/tourniquet inflation	51 (42-62)	56 (47-66)	0.250*
From AI to IVL	28 (26-31)	32 (26-35)	0.280*
From T_2_ to T_3_	21 (12-32)	26 (17-36)	0.250*
**Administered during surgery**			
Mean propofol (s.d.), mg	566 (121)	601 (132)	0.173†
Median remifentanil (i.q.r.), µg	588 (481-740)	622 (530-747)	0.217*
Median sufentanil (i.q.r.), µg	40 (35-45)	40 (35-40)	0.200*
**No. of people using vasoactive drugs**			
Atropine	10 (20.8)	8 (16.6)	0.601†
Ephedrine	14 (29.2)	20 (41.6)	0.200†
Esmolol	0 (0)	2 (4.2)	0.475‡
Nicardipine	0 (0)	3 (6.3)	0.241‡
**Median RSS (i.q.r.)**	2 (2-2)	2 (2-2)	0.618*

Values are *n* (%) unless otherwise indicated. L, lidocaine; N, normal saline; ACLR, anterior cruciate ligament reconstruction; AI, anesthetic induction; IVL, intravenous lidocaine; T_2_, 30 min after tourniquet was inflated; T_3_, the surgery ended or the tourniquet was deflated; RSS, Ramsay sedation score; i.q.r., interquartile range. *Analysed using Mann–Whitney *U* test. †Analysed using independent sample *t* test. †Analysed using χ^2^ test. ‡Analysed using continuity correction χ^2^ test.

### Outcomes

Compared with the N group, the incidence of TH in the L group was significantly decreased (37.5 per cent (18 of 48) *versus* 68.8 per cent (33 of 48); *P* = 0.002). No serious adverse events were observed. The main adverse events after surgery were PONV, dizziness, hypotension and urination pain. There was no difference between the two groups (*Table 2*).

**Table 2:** Tourniquet hypertension and adverse reactions

Outcomes/characteristics	L group (*n* = 48)	N group (*n* = 48)	*P*
Tourniquet hypertension	18 (37.5)	33 (68.8)	0.002†,§
PONV	3 (6.3)	5 (10.4)	0.709‡
Dizziness	5 (10.4)	6 (12.5)	0.744†
Postoperative hypotension	2 (4.2)	0 (0)	0.475‡
Urodynia	1 (2.1)	1 (2.1)	1.000‡
Use of rescue analgesics	0 (0)	2 (4.2)	0.925‡
Use of rescue antiemetics	1 (2.1)	1 (2.1)	0.475‡
Mean (s.d.) duration of stay, h	24.6 (1.6)	24.1 (1.8)	0.153*

Values are *n* (%) unless otherwise indicated. L, lidocaine group; N, normal saline group; PONV, postoperative nausea and/or vomiting.

* Analysed using independent-sample *t* test. †Analysed using χ^2^ test. ‡Analysed using continuity correction χ^2^ test. §Compared with N group, *P* < 0.05.

**Fig. 2:**
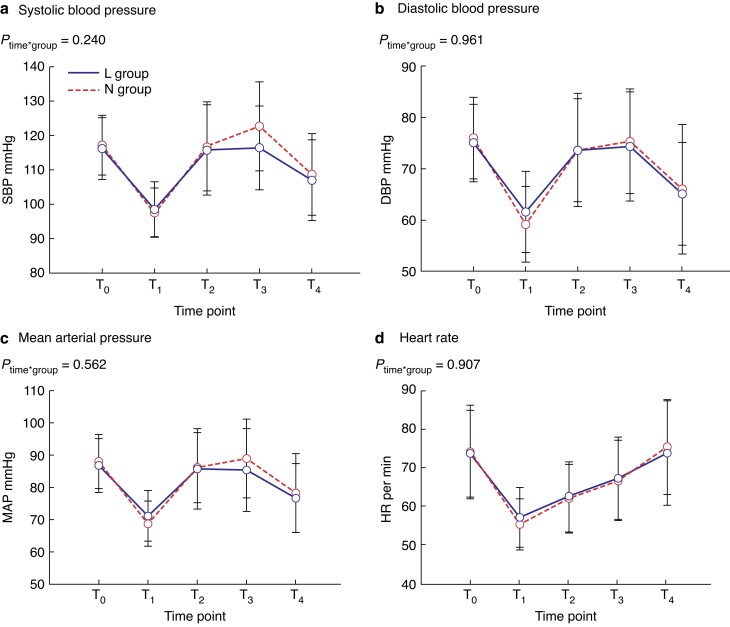
Haemodynamic characteristics **a** Systolic blood pressure. **b** Diastolic blood pressure. **c** Mean arterial pressure. **d** Heart rate. DBP, diastolic blood pressure; HR, heart rate; L group, lidocaine group; MAP, mean arterial pressure; N group, normal saline group; SBP, systolic blood pressure; T_0_, entering the operating room; T_1_, 2 min after the tourniquet was inflated; T_2_, 30 min after the tourniquet was inflated; T_3_, the surgery ended or the tourniquet was deflated; T_4_, 5 min after the tourniquet was deflated.

**Fig. 3:**
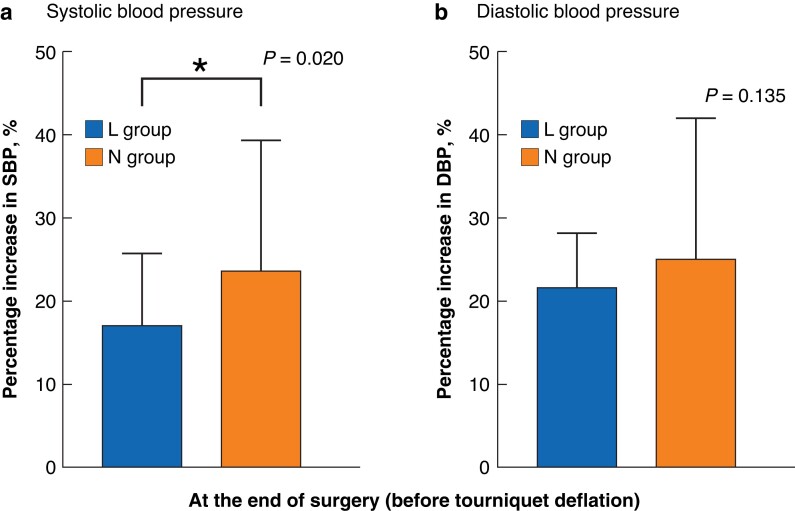
The degree of elevation of blood pressure DBP, diastolic blood pressure; L group, lidocaine group; N group, normal saline group; SBP, systolic blood pressure; *compared with N group, *P* < 0.05.

Throughout the study interval, the SBP, DBP or MAP were not significantly different at any time points between the two groups (*Fig. 2*). However, the degree of elevation of SBP at the end of surgery in the L group was significantly lower than that in the N group (17.1 per cent (8.6-25.8) *versus* 23.6 per cent (12.8-39.3); *P* = 0.020). No significant difference was seen in the degree of elevation of DBP between the two groups (21.5 per cent (10.0-28.0) *versus* 25.0 per cent (18.0-41.75); *P* = 0.135) (*Fig. 3*).

The plan was to draw blood samples from all enrolled patients, however, some patients declined to have blood drawn or missed some time points of blood samples. In addition, some samples were haemolysed. Finally, there were 26 patients with a full set of blood samples for analysis of IL-6 and TNF-α. No difference was found in the concentration of IL-6 between the two groups at T_2_ or T_4_. No difference was found in the concentration of TNF-α between the two groups at T_2_. However, the concentration of TNF-α in the L group at T_4_ (5 min after the tourniquet was deflated) was significantly lower than that in the N group (32.12 pg/ml (22.95-37.94) *versus* 39.89 pg/ml (31.70-53.43); *P* = 0.029) (*Table 3*).

**Table 3:** Inflammatory factors in venous blood

	Median IL-6 (i.q.r.) (pg/ml)			Median TNF-α (i.q.r.) (pg/ml)		
	L group (n = 13)	N group (n = 13)	*P*	L group (n = 13)	N group (n = 13)	*P*
T_5_	7.71 (3.40-18.49)	9.04 (5.75-13.12)	0.687*	7.70 (3.85-12.19)	11.61 (5.69-21.08)	0.125*
T_2_	5.63 (4.16-12.74)	5.42 (4.71-12.38)	0.920*	46.22 (23.04-52.99)	30.56 (22.72-44.61)	0.362*
T_4_	6.58 (4.44-8.34)	6.08 (5.01-10.30)	0.418*	32.12 (22.95-37.94)	39.89 (31.70-53.43)	0.029*,†

L, lidocaine; N, normal saline; IL-6, interleukin 6; TNF-α, tumour necrosis factor-α; T_5_, before anaesthesia induction; T_2_, 30 min after tourniquet was inflated; T_4_, 5 min after the tourniquet was deflated; i.q.r., interquartile range.

* Analysed using Mann–Whitney *U* test. †Compared with N group, *P* < 0.05.

**Fig. 4:**
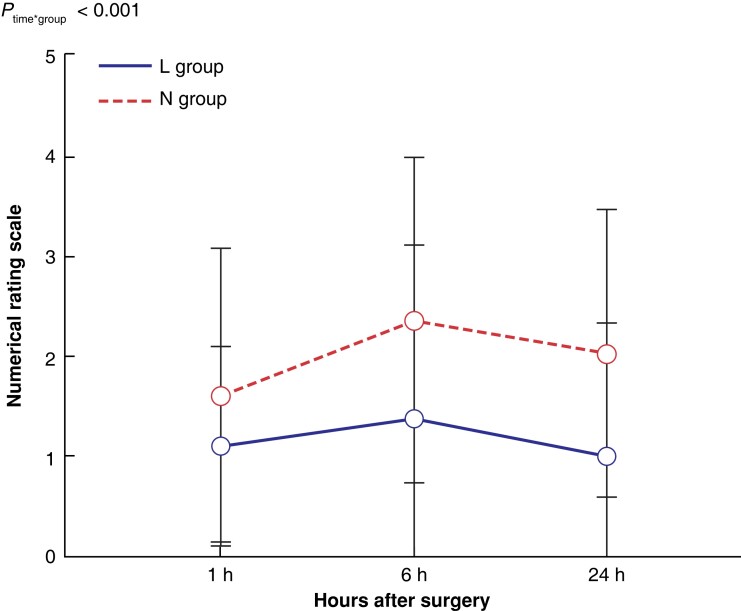
Postoperative pain scores Numerical rating scale: 0, no pain; 10, the most severe pain. L group, lidocaine group; N group, normal saline group.

The NRS of the L group was significantly lower than that of the N group (*P* < 0.001) (*Fig. 4*). No significant difference was found in NRS between the two groups 1 h after surgery (*P* = 0.128). However, the NRS of the L group was significantly lower than that of the L group 6 and 24 h after surgery (*P* = 0.003, *P* < 0.001 respectively) (*Table 4*). In the L group, the total QoR-15 was significantly increased (131 (127-135) *versus* 128 (123-131); *P* = 0.017). In the five dimensions of QoR-15, it was mainly reflected in the improvement of pain (19 (18-20) *versus* 18 (17-19); *P* = 0.004) and no significant difference was found in the other four dimensions (*Table 4*).

**Table 4:** Perioperative pain and quality of recovery

Outcomes/characteristics	L group (*n* = 48)	N group (*n* = 48)	*P*
**NRS**			
Median preoperation (i.q.r.)	3.5 (2.0-5.0)	3.5 (2.0-5.0)	0.852*
Median 1 h postoperation (i.q.r.)	1.0 (0-2.0)	1.0 (0.3-2.8)	0.128*
Median 6 h postoperation (i.q.r.)	0 (0-2.0)	3.0 (1.0-3.0)	0.003*,‡
Median 24 h postoperation (i.q.r.)	0 (0-2.0)	2.0 (1.0-3.0)	<0.001*,‡
**Preoperative QoR-15**			
Median physical comfort (i.q.r.)	46 (44-48)	47 (45-49)	0.075*
Median physical independence (i.q.r.)	18 (17-20)	19 (18-20)	0.473*
Median psychological support (i.q.r.)	20 (18-20)	20 (18-20)	0.932*
Median emotional state (i.q.r.)	35 (33-37)	36 (34-37)	0.090*
Median pain (i.q.r.)	17 (15-18)	17 (15-19)	0.476*
Mean total QoR-15 score (s.d.)	134.2 (6.4)	136.2 (6.2)	0.106†
**Postoperative QoR-15**			
Median physical comfort (i.q.r.)	46 (44-48)	45 (43-47)	0.155*
Median physical independence (i.q.r.)	12 (11-14)	12 (11-12)	0.190*
Median psychological support (i.q.r.)	18 (17-18)	17 (17-18)	0.322*
Median emotional state (i.q.r.)	36 (35-38)	36 (34-37)	0.329*
Median pain (i.q.r.)	19 (18-20)	18 (17-19)	0.004*,‡
Median total QoR-15 score (i.q.r.)	131 (127-135)	128 (123-131)	0.017*,‡

L, lidocaine; N, normal saline; NRS, numerical rating scale; QoR-15, 15-item quality of recovery; i.q.r., interquartile range. *Analysed using the Mann–Whitney *U* test. †Analysed using the independent-sample *t* test. ‡Compared with N group, *P* < 0.05.

## Discussion

This randomized, double-blind, placebo-controlled study showed a significant improvement in TH in patients undergoing ambulatory knee arthroscopy under general anaesthesia who received a single dose of IVL compared with 0.9 per cent normal saline. It was also found that IVL had beneficial effects on postoperative pain and quality of recovery at 24 h after surgery. In addition, IVL could reduce the haemodynamic changes and the release of TNF-α during the perioperative interval.

During knee arthroscopic surgery, patients often experience an increase in BP, with or without increased HR, as the tourniquet on the affected thigh fills and for longer intervals of time. Deepening the level of anaesthesia does not have a good effect on this haemodynamic change. Usually, as the surgery progresses, the level of anaesthesia increases over time, and the pathological autonomic response to the tourniquet persists until the tourniquet is deflated^6,8,25^. Therefore, there is no doubt that the use of a tourniquet is the precipitating factor of TH and the incidence and severity of TH cannot be ignored. Previous studies^7,26^ have found that even in healthy patients, transient and significant haemodynamic changes can cause systemic circulatory effects including increases in central venous pressure, systemic vascular resistance and intracranial pressure, and increase cardiac work. TH also causes additional carbon dioxide load and ventilation load, increases the risk of hypercapnia, then further aggravates the cardiac and cerebral blood volume load. Haemodynamic-related adverse reactions are more likely to occur in patients with cardiovascular diseases (CVD) and obesity^27,28^. This study was designed to avoid these confounding factors to explore the occurrence of TH, so patients were selected who were generally in good condition, with ASA I–II, and excluded vascular diseases and CVD.

The pathophysiology of TH remains unclear. There are currently two potential mechanisms to support the development of TH. First, the metabolic disturbance caused by tourniquet compression is the activation of the autonomic sympathetic nervous system, leading to increased pain and sympathetic impulses, the release of large amounts of plasma catecholamine levels, which in turn leads to increased HR and BP^6,29^. The second mechanism, tourniquet-induced pathophysiological responses, which are chronic pain responses, are mediated by unmyelinated, slowly conducting C-fibres that trigger activation of *N*-methyl-D-aspartate (NMDA) receptors. Physiologically, C-fibres are usually inhibited by rapid pain pulses mediated by myelinated A-δ fibres. Thirty minutes after the tourniquet is inflated, the mechanical compression of the tourniquet results in the blocking of large A-δ fibres, while the C-fibres could still work normally, causing the activation of NMDA receptors, which could lead to the increase of spontaneous firing activity of unresponsive spinal dorsal horn neurons and the expansion of pain receptive fields, resulting in pain hypersensitivity (PH)^7,14,29^. The study chose a single dose of IVL as an intervention primarily to target the second mechanism. IVL has an effect on acute and chronic neuropathic pain and inflammation, reducing spontaneous pain, abnormal pain or PH^17,30^. In addition, surgical trauma, anaesthesia inhibition and tourniquet-induced limb ischaemia-reperfusion can promote an inflammatory response. Inflammation is involved in the induction and maintenance of chronic pain, inflammatory factors drive the inflammatory signalling cascade and promote the activation of nociceptors^15,16,31^. So it was further hypothesized that inflammation plays an important role in the formation of TH.

Dewinter *et al.*^32^ and Hanson *et al.*^33^ both compared the analgesic effect of IVL with that of local block and found that IVL had an equally good postoperative analgesic effect. A double-blind study^34^ observed that lidocaine 10 mg/ml (bolus: 1.5 mg/kg; infusion: 1.5 mg per kg per h) significantly reduced pain and opioid consumption after abdominal hysterectomy. In this study, a single dose of IVL was selected at a concentration of 1.6 per cent (a dose of 1.5 mg/kg diluted to 10 ml with 0.9 per cent normal saline), given 10 min before the tourniquet was inflated. In chronic pain (or TH), the results showed that a single dose of IVL reduced the incidence of TH by 31 per cent compared with the control group. This shows IVL can reduce the PH and the occurrence of spontaneous pain, reduce the series of pathophysiological reactions from tourniquet mechanical pressure, thus inhibiting the occurrence of TH. In postoperative acute pain, the postoperative NRS was significantly lower than that of the control group over time, especially at 6 and 24 h after surgery, and there were fewer requests for remedial analgesia.

Regarding the quality of recovery, a meta-analysis^35^ reported that IVL improves the quality of postoperative recovery by using a validated subjective tool and reduces intraoperative remifentanil consumption. QoR-15 was selected to evaluate the quality of recovery at 24 h after surgery in this study. Although a single dose of IVL did not reduce the consumption of opioids while undergoing surgery, the total score of QoR-15 increased by about 3 points by significantly improving the pain (the remaining four dimensions were not different). Eight cases of PONV were noted and no serious adverse events were observed during the study. Exploring the effect of IVL on PONV was limited by the low incidence of PONV in ambulatory knee arthroscopy^36^.

The role and underlying mechanism of lidocaine in inhibiting neuroinflammation in neuropathic pain have not been fully revealed. Zheng Y *et al.*^37^ studied the effect of lidocaine on the suppressors of cytokine-signalling protein 3 (SOCS3) in rat microglia, showing that lidocaine could alleviate neuropathic pain induced by chronic constriction injury and inhibit the increase of IL-6 and IL-1β. IVL can also inhibit the initiation of peripheral polymorphonuclear cells or neutrophils and reduce TNF-α release^17^. Thus, the study measured the concentrations of IL-6 and TNF-α, and found that a single dose of IVL reduced TNF-α release 5 min after tourniquet deflation. This finding supports the hypothesis that IVL plays an important role in inhibiting TH formation by reducing inflammatory cytokine release; however, no difference in IL-6 was observed in this study. A previous study of dexmedetomidine on oxidative stress after lower extremity ischaemia-reperfusion in unilateral knee replacement, which measured IL-6 at 1 h after tourniquet release, also showed no difference^38^. This may be related to the different time window when inflammatory factors are released. IL-6 increased slowly after trauma and then decreased gradually, usually taking several hours to one day to reach a peak. TNF-α showed a trend of a sharp increase and rapid decrease, and reached a peak within 3 h^39,40^. The effect of a single dose of IVL on IL-6 could not be observed because the tourniquet was not used for more than 1.5–2 h^7,8^, and inflammatory factors were measured 5 min after the tourniquet was deflated (short reaction time window). This study suggests that TNF-α is a better indicator for the early detection of inflammatory response undergoing TH.

The opioid drugs used in this study were remifentanil and sufentanil, the latter is a long-acting anaesthetic and its half-life is shorter, so the time for anaesthesia and sedation is shorter. However, sufentanil is more liposoluble and reaches the central nervous system directly through the blood brain barrier so it has a stronger analgesic effect. A previous study^41^ showed that sufentanil would not have a greater impact on the haemodynamics of patients, which was also helpful when observing the haemodynamic effects in this study. The combination of the two drugs can reduce the administration of anaesthetic drugs in general anaesthesia surgery, avoid the occurrence of adverse reactions after surgery and greatly improve the analgesic effect of anaesthesia. At the same time, they can effectively maintain the stability of patients’ haemodynamics, shorten the time for patients to wake up from anaesthesia, reduce restlessness during waking and help patients recover quickly^42^. Although there were no differences in intraoperative SBP, DBP, MAP and HR between the two groups, a single dose of IVL reduced the increase of SBP by about 6.5 per cent, which demonstrated the effectiveness of IVL in reducing TH incidence. TH was defined as an SBP or DBP ≥ 30 per cent higher than baseline. Therefore, by observing the degree of haemodynamic increase or decrease over a certain interval of time during the perioperative interval, compared with the intuitive haemodynamic measurement value, the occurrence of TH can be more effectively and accurately captured, and the risk of pathological injury and related complications can be reduced in a timely manner.

This study has several limitations. First, the trial was not a multicentre study, which may make the results not universal. Second, a single dose of IVL was selected in this study, without the combination of the continuous intravenous infusion of lidocaine. In the future, it can be further explored whether a single dose of IVL combined with an infusion of IVL could bring more beneficial effects to patients undergoing arthroscopy. Third, assessments of long-term pain and functional outcomes such as postoperative vascular, nerve, muscle injuries are missing. Larger studies are needed to observe long-term outcomes in patients undergoing ambulatory arthroscopy.

## Data Availability

The data sets of the present study are available from the corresponding author upon reasonable request.
